# Editorials

**Published:** 1890-11

**Authors:** 


### HOMŒOPATHIC PHYSICIAN,

A MONTHLY JOURNAL OF

HOMŒOPATHIC MATERIA MEDICA AND CLINICAL MEDICINE.

"If our school ever gives up the strict inductive method of Hahnemann, we are lost, and deserve only to be mentioned as a caricature in the history of medicine."—CONSTANTINE HERING.

Vol. X. NOVEMBER, 1890. No. 11.

### EDITORIAL.

MEDICINAL AGGRAVATION.—"An aggravation of the disease by new, violent symptoms during the first few doses of a chosen curative medicine is *never* indicative of feebleness of the dose (never requires the dose to be increased), but it proves the total unfitness and worthlessness of the medicine in this case of disease.

"The aggravation just alluded to, by violent, new symptoms not proper to the disease, bears no resemblance to the increase of the apparently original symptoms of the disease during the first few hours after the administration of a medicine selected in a positive (curative) manner, which I formerly spoke of. This phenomenon of the increase of what seems to be the pure symptoms of the disease, but which are actually predominant medicinal symptoms resembling those of the disease, indicates merely that the dose of the appropriately selected curative medicine has been too large. It disappears, if the dose has not been enormously large, after the lapse of two, three, or, at most, four hours after its administration, and makes way for a removal of the disease that will be all the more durable, generally after the expiring of the term of the action of the first dose; so that, in the case of acute affections, a second dose is usually unnecessary.

"However, there is no positive remedy, be it ever so well selected, which shall not produce one, at least one slight, unusual suffering, a slight new symptom, during its employment, in very irritable, sensitive patients, for it is almost impossible that medicine and disease should correspond as accurately in their symptoms as two triangles of equal angles and sides resemble each other. But this unimportant difference is (in favorable cases) more than sufficiently compensated by the inherent energy of the vitality, and is not even perceived, except by patients of excessive delicacy."—*Hahnemann.*

This question of aggravation, treated by Hahnemann above, is one that has received as much ridicule from the old-school and from many of his pretending followers as any of the other truths he promulgated. It requires but slight observation on the part of him who knows how to practice Homœopathy to know that it is true, and that it is but a part of the duty of the conscientious physician to be ever on the alert for aggravations, so that he may know how closely his chosen remedy fits the condition for which it is prescribed.

To fit himself for the ability to notice the changes which occur in the treatment of any case of disease is incumbent upon every one, and is one of the fundamental doctrines of Homœopathy.

It is here a knowledge of real pathology is necessary. We do not mean that coarse, material pathology usually taught in the schools, but that finer study which is to be found alone in the writings of Hahnemann and his followers. This knowledge, as there found, enables the genuine physician to know when to give and when to withold medicine, and the successful treatment of disease can only be attained by adhering to this.

---

In this, as in all other of his writings, Hahnemann does not explain, or attempt to explain, the why. He possessed sufficient wisdom to be satisfied with observing and recording the facts. It has remained for the present day to produce wiser men than Hahnemann.

Our attention is frequently called to papers in which the writers endeavor to comment on selected paragraphs of the *Organon*, and we are more than amused by their absurd attempts to pose as erudite.

Hahnemann recorded the facts. These writers make frivolous attempts at philosophical explanations of them, or else equally frivolous attempts to deny them.

If there be no explanation that satisfies their so-called "understanding," then they deny the facts. Our literature teems with this sort of rubbish. The same energy thus expended, if used to carefully verify Hahnemann's facts and amplify his *Materia Medica*, would add to the progress of the cause.

That principle which we call *dynamis* is a favorite subject of explanation or of attack according as the writer is in sympathy with pure Homœopathy or in a state of hostility to it.

The attempt to explain it reminds us of the student who had known, but, unfortunately, had forgotten what is animal magnetism.

Lo, here are greater than the master!
"A strong conceit is rich; so most men deem,
If not to be, 'tis comfort yet to seem."

G. H. C.

---

THE TEACHING OF PURE HOMŒOPATHY IN THE COLLEGES.—In our October number, at page 437, we spoke of the appointment of Dr. Frank Kraft to the chair of Materia Medica in the Cleveland College. In that editorial we unintentionally overlooked Dr. W. L. Reed, of St. Louis, who is doing an equally noble work, in the teaching of pure Homœopathy in the St. Louis College.

In a letter received from Dr. Cohen, of Waco, Texas (which much to our regret we cannot publish in full for want of space), he writes: "Dr. Reed lectures on Materia Medica three times per week, and teaches his auditors how to apply the remedies under the strictest observance of the law. The classes read the *Organon* twice a week, the Doctor commenting upon each paragraph at length, and he has succeeded in exciting an intense interest on the subject, in the minds of his pupils. I was in St. Louis in March last and know whereof I write. There are other gentlemen in this faculty who are just as zealous and painstaking in their respective spheres in disseminating the true principles which should guide us in our practice, as is Dr. Reed, and it is due to these hard workers in the cause that every practitioner and every patron of Homœopathy should know what is being done at the institution in St. Louis, and permit it at least to share in the good things said of the Cleveland school."

Dr. Reed is sufficiently well acquainted with us to know that we would not intentionally ignore his work. Still we think it only right that we should make this explanation that no injustice be done a most enthusiastic, painstaking advocate of the cause.

